# Successful Prolonged Desensitization to Epoetin-Alfa Hypersensitivity in an Adult Patient With Advanced Chronic Kidney Disease

**DOI:** 10.7759/cureus.40424

**Published:** 2023-06-14

**Authors:** Hemali Panchal, Saiyed Ali, Rosemary Hallett, Brian Young, Nasim Wiegley

**Affiliations:** 1 Internal Medicine, University of California Davis School of Medicine, Sacramento, USA; 2 Nephrology, University of California Davis School of Medicine, Sacramento, USA; 3 Allergy and Immunology, University of California Davis School of Medicine, Sacramento, USA

**Keywords:** drug-induced hypersensitivity, synthetic epo, desensitization therapy, chronic kidney disease (ckd), anemia of chronic diseases

## Abstract

The use of erythropoiesis-stimulating agents (ESAs) reduces the need for recurrent blood transfusions in patients with advanced kidney disease. Rarely, allergic reactions to recombinant human erythropoietin can develop, complicating anemia management due to cross-reactivity between these agents. We report the use of an outpatient desensitization protocol, which was successfully completed in an adult patient who developed a maculopapular rash as a form of delayed-type hypersensitivity reaction (DTH) to epoetin-alfa (EPO) use, followed by successful re-introduction of EPO and continued tolerance.

## Introduction

Recombinant human erythropoietin is used widely in patients with anemia associated with chronic kidney disease (CKD). Treatment of anemia due to CKD is important for several reasons, including improvement in quality of life, reducing hospitalizations and/or hospital length of stay [1], risk of cardiovascular disease [2], and the need for blood transfusions, which can lead to volume disturbances, iron overload, and preformed antibodies complicating future organ transplants [3]. Allergic reactions to epoetin-alfa have been reported in the literature rarely. Reactions include orofacial angioedema and anaphylaxis leading to shock [4,5], which are types of immediate-type hypersensitivity, and exanthematous pustulosis [6], which is a type of delayed hypersensitivity reaction.

In previously reported cases of adult patients with hypersensitivity reactions to ESAs, inpatient desensitization protocols were performed, which resulted in successful reintroduction to these agents [5,6]. To the best of our knowledge, an outpatient desensitization protocol has only been described in a pediatric patient in the past [7] We present a case of an adult patient with evidence of a delayed-type hypersensitivity drug reaction to epoetin-alfa who underwent a successful prolonged outpatient desensitization protocol.

## Case presentation

**Figure 1:**
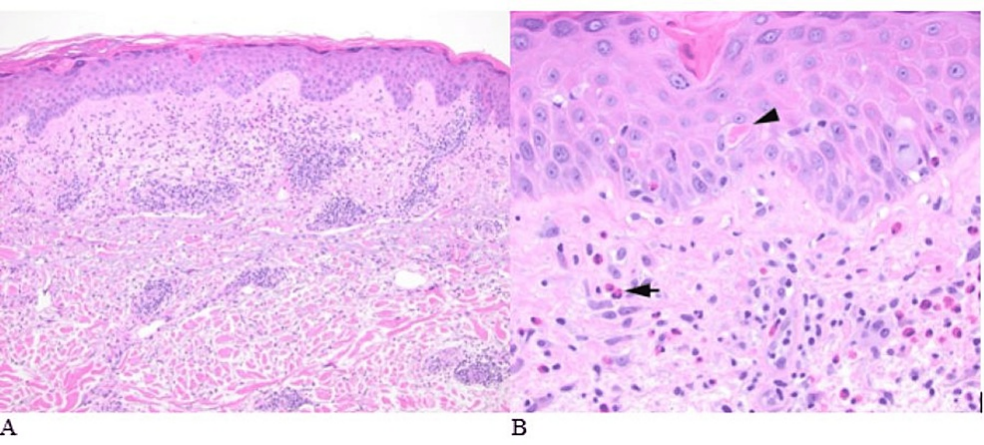
Histopathologic features of the cutaneous eruption were consistent with a drug reaction Note the eosinophilic spongiosis, dyskeratotic keratinocytes (arrowhead), and perivascular and interstitial eosinophils (arrow). (Hematoxylin-eosin stain; original magnifications: A, ×100; B, ×400)

A white man in his 70s developed anemia in the setting of chronic kidney disease stage IV secondary to diabetes mellitus. He was initiated on epoetin-alfa (EPO) subcutaneous (SC) 10,000 units per month for the management of chronic anemia. After the first dose, he developed a maculopapular rash in the back and chest, which was not initially reported to his medical providers. He received three doses of SC EPO about one month apart, with slow improvement in hemoglobin levels; however, each dose was accompanied by a progressively worsening mildly pruritic rash shortly after EPO administration, starting at the injection site and spreading to his back and extremities, with pink minimally scaly papules coalescing into plaques. He did not have mucosal involvement. He denied the use of any new medications, topical creams, or chemical exposures. The decision to hold additional doses of EPO therapy was made to allow further evaluation and safe management. He underwent a skin biopsy, which showed perivascular and interstitial infiltrates of eosinophils, interface alteration with dyskeratotic keratinocytes, and spongiosis, supporting a drug-mediated hypersensitivity reaction (Figure 1).

In the setting of the known risk of cross-reactivity between various erythropoietin-based molecules, additional formulations, such as darbepoetin alfa, were not trialed [8,9]. Furthermore, non-erythropoietin drugs, such as those that influence the hypoxia-inducible factor pathway, have not yet been Food and Drug Administration (FDA)-approved for use in pre-dialysis settings. He was subsequently referred to an allergist for the development and administration of an individualized EPO desensitization protocol. Based on the subacute nature of his symptoms, he underwent a 17-day prolonged desensitization protocol in the outpatient setting, followed by a successful re-introduction of EPO (Table 1). He continued to tolerate subcutaneous EPO administrations effectively for more than two years without any reactions.

**Table 1:** Desensitization protocol, with doses adjusted to reflect adult dosing Modified from Rosa et al. [7]

Desensitization protocol, with dosages modified to reflect adult dosing	
Day	Dose (IU)
1	50
1	100
4	200
8	400
11	800
15	1600
18	3200
22	6400

## Discussion

Currently, ESAs remain the treatment of choice for anemia of CKD to limit the need for blood transfusions. Thus, an ESA allergy creates a challenge in kidney disease care, and it can manifest in several ways. Immediate hypersensitivity reactions, such as hives or anaphylaxis, typically develop within minutes to hours after exposure and are mediated by immunoglobulin E (IgE) antibodies. In contrast, delayed-type hypersensitivity reactions (DTH) emerge later and usually take 24 to 72 hours to manifest. DTH reactions involve T cells and cytokines, leading to inflammation and tissue damage. While immediate reactions are rapid and potentially life-threatening, delayed reactions are characterized by delayed onset but can be equally bothersome and persistent [10]. Although polysorbate, an emulsifier used in some pharmaceutical agents, has been thought to play a role in triggering the immediate hypersensitivity reaction to ESA agents [11,12], the exact trigger for the DTH reactions is less clear. Cross-reactivity has been described, thus changing the formulation is not routinely recommended [8,9].

Prior reported cases of adult patients with allergic reactions to ESA therapy have been mostly associated with immediate-type hypersensitivity to ESA. These patients underwent successful desensitization with rapid three-hour to three-day inpatient protocols, depending on the severity of symptoms. In the case of anaphylactic shock to ESA, a three-hour protocol in the intensive care unit was used [5]. In the case of exanthematous pustulosis, a three-day protocol was used [6].

Based on the stable clinical status of our patient and the subacute nature of his symptoms, we decided to proceed with the development of a prolonged outpatient desensitization protocol adapted from the Rosa protocol [7], with modified dosing required for an adult patient. The patient tolerated the desensitization without incident. He was monitored for two hours after each dose in the outpatient Allergy and Immunology Clinic, with standard treatment for anaphylaxis readily available (including epinephrine, oxygen, intravenous fluids, and antihistamines). He did not require the use of any as-needed treatments for allergic reactions. The patient continued to tolerate subcutaneous EPO administration for two years, with successful anemia management, and without any recurrence of the symptoms. He later progressed to requiring dialysis initiation and transitioned to intravenous EPO with continued tolerance.

## Conclusions

This case demonstrates the ability to safely deliver desensitization to erythropoietin-stimulating agents in the outpatient setting for patients with subacute symptoms and stable clinical status, eliminating the need for hospitalization and escalation of care. The desensitization protocol should be modified for each patient based on the severity of the symptoms to deliver individualized care. Outpatient desensitization should be considered in those with non-life-threatening delayed-type hypersensitivity reactions to epoetin alfa, as it remains the mainstay of treatment for chronic anemia due to advanced kidney disease.

## References

[1] Jones M, Ibels L, Schenkel B, Zagari M (2004). Impact of epoetin alfa on clinical end points in patients with chronic renal failure: a meta-analysis. Kidney Int.

[2] Cases A, Coll E, Collado S (2009). Anemia in chronic kidney disease and its cardiovascular implications (Article in Spanish). Med Clin (Barc).

[3] Fishbane S, Spinowitz B (2018). Update on anemia in ESRD and earlier stages of CKD: core curriculum 2018. Am J Kidney Dis.

[4] García JE, Senent C, Pascual C (1993). Anaphylactic reaction to recombinant human erythropoietin. Nephron.

[5] Aziz N, Luna C, Mirza F, Tobin M (2015). Anaphylactic shock at the end of hemodialysis. Semin Dial.

[6] Ruano FJ, Garcimartin MI, Vazquez de la Torre M, Blanca M, Canto G (2009). Desensitization of epoetin-alpha in a confirmed case of acute exanthematic pustulosis. Allergy.

[7] Rosa JS, Vuong VB, Haskin O, Liu AY (2018). A novel outpatient desensitization protocol for recombinant human erythropoietin allergy in a pediatric patient. Allergy Asthma Clin Immunol.

[8] Rueangsri R, Laisuan W (2020). Epoetin-alfa induced pruritic maculopapular eruption: case report and literature review. Asian Pac J Allergy Immunol.

[9] Weber G, Gross J, Kromminga A, Loew HH, Eckardt KU (2002). Allergic skin and systemic reactions in a patient with pure red cell aplasia and anti-erythropoietin antibodies challenged with different epoetins. J Am Soc Nephrol.

[10] Apicella MA, Allen JC (1969). A physiologic differentiation between delayed and immediate hypersensitivity. J Clin Invest.

[11] Limaye S, Steele RH, Quin J, Cleland B (2002). An allergic reaction to erythropoietin secondary to polysorbate hypersensitivity. J Allergy Clin Immunol.

[12] Steele RH, Limaye S, Cleland B, Chow J, Suranyi MG (2005). Hypersensitivity reactions to the polysorbate contained in recombinant erythropoietin and darbepoietin. Nephrology (Carlton).

